# Role of Epigenetic Modifications in Pathology of COPD

**Published:** 2017

**Authors:** Ian Michael Adcock

**Affiliations:** National Heart & Lung Institute (NHLI), Imperial College London

The epigenome is a set of heritable modifications and tags that affect the genome without changing the intrinsic DNA sequence. These marks include DNA methylation, modifications to histone proteins around which DNA is wrapped and expression of noncoding RNA. Alterations in all of these processes have been reported in patients with COPD. In some cases these differences are linked to disease severity and susceptibility and may account for the limited value of genetic studies in COPD. Animal models of COPD suggest that epigenetic modifications and processes are linked to COPD and may be tractable targets for therapeutic intervention

